# Impact of Non-viral Coinfections on Mortality of Severely Ill COVID-19 Patients in Dubai

**DOI:** 10.7759/cureus.26977

**Published:** 2022-07-18

**Authors:** Rashid Nadeem, Aju Rafeeq, Anas A Aga, Ayesha Siddiqua, Ekta Sharma, Doaa Anwer, Mohd Kafeel Khan, Mohamed Abdulla Mohammed Hussein, Yusra Omar Alshaikh SayedAhmed, Farooq Ahmad Dar

**Affiliations:** 1 Intensive Care Medicine, Dubai Hospital, Dubai, ARE; 2 Medicine, Dubai Health Authority, Dubai, ARE; 3 Anesthesiology, Dubai Hospital, Dubai, ARE; 4 Intensive Care Unit, Dubai Hospital, Dubai, ARE; 5 Pediatric Critical Care Medicine, Dubai Hospital, Dubai, ARE; 6 Pediatrics Medicine, Dubai Hospital, Dubai, ARE; 7 Cardiac Surgery, Dubai Health Authority, Dubai, ARE

**Keywords:** length of stay in icu, length of stay in the hospital, mortality, secondary bacterial infection, covid-19

## Abstract

Background: Coronavirus disease 2019 (COVID-19) infections may have been associated with secondary infection. Community-acquired or hospital-acquired such infections affect clinical outcomes. We performed a retrospective study to evaluate the impact of these infections on clinical outcomes.

Methods: This was a retrospective analysis of all consecutive patients with COVID-19 admitted to the intensive care unit (ICU) of Dubai hospital.

Results: Patients with secondary non-viral infections (SNIs) have higher mortality than patients without SNIs (57.3% vs. 43.7%, p=0.037). Patients with SNIs had more days on mechanical ventilation (MV) 19(11-27) vs. 5(2-10) p<0.001, more LOSICU 22 (15-33) vs. 7 (2-11) p<0.001, and more length of stay in hospital (LOSH) 28 (18-45) vs. 11.5 (6-19), p<0.001. Multiple logistic regression analyses showed that SNIs do not predict mortality. Linear logistic regression analysis showed patients with SNIs have increased length of stay in ICUs (LOSICUs), length of stay in hospitals (LOSHs), and prolonged needs for MV.

Conclusion: SNIs are high in patients admitted to ICU for COVID-19 acute respiratory distress syndrome (ARDS). Although they do not impact mortality, they prolong the need for MV, LOSICU, and LOSH.

## Introduction

Coronavirus disease 2019 (COVID-19), also known as severe acute respiratory syndrome coronavirus 2 (SARS-CoV-2), caused a pandemic starting in Wuhan, China, in December 2019. In unfortunate patients, it may lead to acute respiratory distress syndrome (ARDS) with a high rate of admission to the intensive care unit (ICU) [1]. Patients admitted to ICU are frequently found to have colonization and coinfection with bacteria and fungi which may increase mortality [2]. A metanalysis documented that about 14% of patients admitted to ICU have coinfection with bacteria or fungi [3]. These patients share many risk factors for secondary non-viral infections (SNIs) including treatment with steroids, urinary catheters, intravascular catheters, endotracheal tubes, and prolonged stay on mechanical ventilation (MV) [4]. The SNIs are associated with high morbidity and mortality [5]. Infection control, prevention, and early treatment of these infections are crucial to improving the outcomes in this patient population. Only a few studies have described SNIs [6-7]. These studies studied patients admitted to the general ward with moderate severity of illness [6-7]. We aimed to study the prevalence of these co-infections and their impact on clinical outcomes in severely sick patients admitted to ICU.

## Materials and methods

We conducted a retrospective analysis of all consecutive patients with COVID-19 admitted to the ICU of Dubai hospital from January 1st, 2020 through June 30th, 2020. Standard protocols regarding infection control and prescription of antimicrobials are followed in our hospital. It includes no antibiotic prophylaxis and application of ventilator-associated pneumonia (VAP) bundles which include a set of procedures to reduce VAP. If a patient is suspected to have coinfection then septic work-up including blood cultures, urine cultures, and pneumonia panels were ordered to gather objective evidence of coinfection. The study was approved by the Dubai Scientific Research Committee. Written informed consent was waived because of the retrospective nature of the analysis. All consecutive patients with laboratory-confirmed SARS-CoV-2 infection (positive reverse transcription polymerase chain reaction results), admitted to the ICU were included. Exclusion criteria were age < 18 years and reason for ICU admission other than COVID-19. The following patient data were collected at admission: demographics; age and body mass index (BMI); comorbidities, immunocompromised status (i.e., chronic immunosuppressive therapy, active hematologic or solid malignancies, autoimmune diseases); diabetes, hypertension; acute physiology and chronic health evaluation (APACHE-2) scores; and blood laboratory results (complete blood count, creatinine, C-reactive protein, D-dimers, ferritin). We also recorded the therapeutic treatment provided; the use of hydroxychloroquine, lopinavir and ritonavir, corticosteroids, and tocilizumab. Specialized therapies were also recorded; continuous renal replacement therapy (CRRT), extracorporeal membrane oxygenation (ECMO), and prone position therapy. The following outcomes were recorded: mortality, length of stay in ICU (LOSICU), length of stay in hospital (LOSH), and duration of MV. Infections were identified by physicians taking care of patients who were intensivists. Secondary coinfections were diagnosed as VAP, hospital-acquired pneumonia (HAP), catheter-associated urinary tract infection (CRUTI), bloodstream infection (BSI), catheter-related bloodstream infection (CRBSI), *Clostridium difficile* colitis, suspected or proven invasive candidiasis, and invasive pulmonary aspergillosis. Viral infections were not recorded. Use of sedation, paralysis, and vasopressors were also recorded.

We defined multi drug resistance (MDR) as all microorganisms resistant to at least one agent in three or more antimicrobial classes of agents [8] or the microorganisms with specific antibiotic resistance mechanisms (i.e., methicillin-resistant Staphylococcus aureus). Each antibiotic susceptibility testing was analyzed, and resistance patterns for each antimicrobial agent were classified. 

Statistical analyses

Descriptive statistics were produced for demographic, clinical, and laboratory characteristics of patients. Mean and standard deviation (SD) (or, in case of skewed distribution, median and interquartile range [IQR]) are reported for continuous variables, and numbers and percentages are reported for categorical variables. Groups are compared with parametric or nonparametric tests, according to data distribution for continuous variables and with the Pearson test (or Fisher exact test when appropriate) for categorical variables. All tests were two-sided, and p value < 0.05 was chosen to indicate statistical significance. We utilized SPSS (IBM Corp. Released 2020. IBM SPSS Statistics for Windows, Version 27.0. IBM Corp., Armonk, NY) for statistical analysis.

## Results

The characteristics of the total sample and two groups (patients with SNIs [cases] and patients without SNIs [controls]) are shown in Table 1 for categorical variables and in Table 2 for continuous variables.

**Table 1:** Sample characteristics (categorical variables). CAD, coronary artery disease; CRRT, continuous renal replacement therapy; ECMO, extracorporeal membrane oxygenation; GI, gastrointestinal prophylaxis

	All patients	Secondary infection	No secondary infection	
Clinical features	N=237	N=119	N= 118	p value
Male (%)	208 (87.8)	108 (90.8)	100 (84.7)	0.158
Cough	190 (80.5)	99 (83.2)	91 (77.8)	0.294
Fever	216 (91.1)	111 (93.3)	105 (89)	0.245
Dyspnea	191 (80.6)	99 (83.9)	92 (77.3)	0200
Gastric symptoms	29 (12.9)	14 (12.6)	15 (13.2)	0.903
Diabetes	103 (43.3)	48 (40.3)	55 (46.2)	0360
Hypertension	59 (25)	28 (23.5)	31 (26.5)	0.599
CAD	16 (6.8)	7 (5.9)	9 (7.6)	0.617
Renal disease	25 (10.8)	15 (13)	10 (8.5)	0.269
Outpatient dialysis	16 (6.7)	13 (10.9)	3 (2.5)	0.010
Immunodeficiency	10 (4.3)	5 (4.3)	5 (4.3)	0.100
Clinical variables				
Inpatient fever	205 (86.5)	108 (90.8)	97 (82.2)	0.054
Tachycardia	187 (78.6)	103 (86.6)	84 (70.6)	0.003
Hypotension	120 (50.4)	76 (63.9)	44 (37)	0.001
Hypoxia	204 (85.7)	109 (91.6)	95 (79.8)	0.010
Mechanical vent	203 (85.3)	111 (93.3)	92 (76.7)	0.001
Vasopressors	187 (78.9)	108 (90.8)	79 (66.9)	0.001
CRRT	73 (30.7)	52 (43.7)	21 (17.6)	0.001
Treatment				
Chloroquine	209 (88.2)	101 (85.6)	108 (90.8)	0.218
Lopinavir/Ritonavir	86 (36.4)	50 (42.7)	36 (30.3)	0.046
Favipiravir	190 (80.2)	97 (82.2)	93 (78.2)	0.434
Steroids	188 (79.3)	106 (89.8)	82 (68.9)	0.001
Tocilizumab	37 (15.7)	24 (20.5)	13 (10.9)	0.043
Tracheostomy	31 (13)	26 (21.8)	5 (4.2)	0.001
ECMO	13 (5.5)	11 (9.2)	2 (1.7)	0.010
Sedatives	211 (88.7)	118 (99.2)	93 (78.2)	0.001
Narcotics	181 (76.7)	100 (76.7)	81 (69.2)	0.007
Paralytics	202 (84.9)	113 (84.9)	89 (74.8)	0.001
Anticoagulation	229 (97)	117 (99.2)	112 (94.9)	0.055
GI prophylaxis	224 (96.6)	115 (99.1)	109 (94)	0.031
Mortality	119 (50.4)	67 (57.3)	52 (43.7)	0.037

**Table 2:** Sample characteristics (continuous variables). IQR, inter quartile range; BMI, body mass index; WBC, white blood cell; CRP, C-reactive protein; CPK, creatinine phosphokinase; MV, mechanical ventilation; ABG, arterial blood gas pH; LOSICU, increased length of stay in ICU; LOSH, length of stay in hospital

Continuous variables	Total N=237	Secondary infection N=119	No secondary infection N=118	
	Median (IQR)	Median (IQR)	Median (IQR)	p value
Age (years)	49 (42-57)	50 (44 -57)	47.0 (41.0-56)	0.105
BMI (kg/m^2^)	27.5 (25-31)	27.5 (25 -31.3)	27.6 (25.0-31)	0.939
WBC (10^3^/microliter)	7.9 (6-10.8)	7.7 (6 -10.7)	8.0 (6.1-11.6)	0.503
Ferritin (ng/mL)	1293 (577-1945)	1270 (706.3 -2131)	1319.5 (470.4-1845)	0.080
D-Dimer (ng/mL)	1.17 (0.57-3.73)	1.21 (0.61 -4.05)	1.1 (0.5-3.3)	0.290
Procalcitonin (ng/mL)	0.34 (0.13-1.09)	0.34 (0.15 -1.16)	0.3 (0.1-1)	0.473
CRP (mg/L)	129 (75-215)	122 (72 -218)	135.0 (77.0-215.8)	0.594
Creatinine (mg/dl)	0.9 (0.8-1.2)	1 (0.8 -1.3)	0.9 (0.7-1.2)	0.016
CPK (units/L)	226 (96-653)	29 (114.5 -887)	175 (79.3-482.8)	0.015
ABG pH	7.38 (7.28-7.43)	7.39 (7.27 -7.44)	7.4 (7.3-7.4)	0.686
PCo2 (Torr)	37.3 (31.3-47.3)	37.7 (30.8 -47.3)	35.4 (31.9-47.5)	0.992
PO2 (Torr)	63 (47-90)	66.9 (50.8 -86.1)	59.8 (45.9-93.6)	0.260
Lactate (mmol/L)	1.7 (1.3-2.5)	1.7 (1.2 -2.7)	1.7 (1.3-2.4)	0.976
Bicarbonate (mEq/L)	21.6 (18.9-24)	22 (19.2 -24.3)	21.2 (18.9-24)	0.458
Magnesium (mg/dL)	2.04 (1.9-2.2)	2.03 (1.9 -2.25)	2.1 (1.9-2.3)	0.338
Platelets (10^3^/microliter)	203 (155-263)	185.5 (146.75 -255.7)	222.0 (172.0-285.5)	0.014
Days on MV	11 (4.2-20.7)	19 (11 -27.2)	5.0 (2.0-10)	<0.001
LOSICU (days)	14 (5-23)	22 (15 -33.2)	7.0 (2.0-11)	<0.001
LOSH (days)	19 (9-32)	28 (18 -45.5)	11.5 (6.0-19)	<0.001
APACHE 2 scores	16 (13-21)	16 (12.7 -20.2)	17.0 (13.0-22)	0.582

Patients with secondary non-viral infections have higher mortality than patients without secondary non-viral infections (57.3% vs 43.7%, p=0.037). Patients with secondary non-viral infections had more days on MV, 19 days (11-27) vs 5 days (2-10), p<0.001, and more LOSICU, 22 days (15-33) vs 7 days (2-11) p<0.001, and more LOSH 28 days (18-45) vs 11.5 days (6-19), p<0.001. Patients with secondary non-viral infections also had a higher occurrence of inpatient tachycardia, hypoxemia, and hypotension upon admission and they were more likely to be treated with vasopressors and mechanical ventilation than patients without secondary non-viral infections (Table 2). Details of the organisms with positive culture results in blood, sputum, and urine are provided in Table 3.

**Table 3:** Microorganisms of the secondary non-viral infection (N=237). MDR, multidrug resistance

Variable	Ventilator associated pneumonia	Blood stream infection	Catheter-related blood stream infection	Urinary tract infection	Overall	MDR
Included patients	72 (30.3)	84 (35.8)	11 (4.6)	36 (15.1)	192 (81)	44 (23)
Gram-staining microorganisms						
Gram-positive microorganisms	22 (30.5)	40 (48)	3 (27)	11 (30)	76 (39.5)	14 (31)
Staphylococcus aureus	14 (19)	11(27)	1 (9)	1 (9)	27 (35.5)	13 (92)
Enterococcus species	3 (4.1)	15 (45)	1 (9)	9 (81)	28 (36.8)	
Coagulase-negative staphylococci	2 (2.7)	9 (22)		1 (9)	12 (15.7)	
Streptococcus pneumoniae	1 (1)	1 (2.5)			2 (2.6)	
Other	2 (2.7)	1 (2.5)	1 (9)		4 (5.2)	1 (8)
Gram-negative microorganisms	57 (79)	50 (59)	8 (72)	25 (69.4)	120 (62.5)	30 (68)
Pseudomonas aeruginosa	23 (40)	9 (18)	3 (37)	3 (12)	38 (31.6)	5 (16)
Enterobacterales (other)	1 (1.7)	5 (10)		2 (8)	8 (6.6)	5 (16)
Klebsiella species	29 (51)	11 (22)	3 (37)	4 (16)	47 (39.1)	7 (23)
Escherichia coli	1(1.7)	9 (18)	2 (25)	16 (64)	28 (23.3)	13 (43)
Acinetobacter baumannii	1(1.7)	7 (14)			8 (6.6)	
Other	3 (5.2)	9 (18)			12 (10)	
Fungi	5 (6.9)	8 (10)	1 (9)			
Candida	4 (80)	8 (100)				
Aspergillus	1 (20)					

We conducted univariate logistic regression to assess the effect of each variable on mortality. Those variables found to be significant on univariate logistic regression were included in the final multiple logistic regression which showed that SNIs do not predict mortality (Table 4).

**Table 4:** Multiple logistic regression predicts of mortality. APACHE, acute physiology and chronic health evaluation

Predictor variables	Beta	Odds ratio	95% CI for odds ratio		p value
			Lower limit	Upper limit	
Age	0.033	1.034	0.988	1.082	0.154
BMI	0.137	1.146	1.047	1.255	0.003
Renal failure	1.168	3.215	0.689	15.003	0.137
Tachycardia	-0.266	0.766	0.243	2.419	0.650
Hypotension	0.701	2.015	0.776	5.236	0.150
Vasopressors	1.482	4.400	0.813	23.797	0.085
Inpatient dialysis	1.210	3.354	1.221	9.214	0.019
Lopinavir/Ritonavir	0.969	2.634	1.056	6.571	0.038
Favipiravir	-0.475	0.622	0.165	2.344	0.483
Steroids	-1.851	0.157	0.041	0.600	0.007
Tracheostomy	-2.620	0.073	0.019	0.272	0.000
Sedatives	0.013	1.013	0.086	11.898	0.992
D-Dimer	0.030	1.031	0.987	1.077	0.176
APACHE 2 score	0.010	1.010	0.941	1.084	0.781
Culture positive	0.121	1.128	0.463	2.749	0.790

For the evaluation of the effect of infections on LOSICU, LOSH, and MV linear regression modeling was performed. For LOSICU, LOSH, and days on MV, we assessed the data distribution and found that data were not normally distributed; therefore, data were transformed into Log (ln) values. Univariate linear regression for each variable was performed and only those variables were included in the final multiple linear regression analysis which showed that patients with SNIs have increased LOSICU (Table 5) and LOSH (Table 6) and prolonged need for MV (Table 7).

**Table 5:** Multiple linear regression for predictors of length of stay in ICU. ICU, intensive care unit

Predictor variables	Unstandardized coefficients	Standardized coefficients	95% confidence interval for odds ratio		p value
	B	Odds ratio	Lower limit	Upper limit	
Gastric complaints	-6.714	-0.141	-11.059	-2.369	0.003
Fever on admission	-0.185	-2.422	-0.336	-0.034	0.016
Hypotension	0.007	0.134	-0.102	0.117	0.893
Ventilation	-0.062	-0.624	-0.257	0.133	0.534
Pressers	0.053	0.669	-0.103	0.209	0.505
Dialysis	0.010	0.172	-0.101	0.120	0.864
Lopinavir/Ritonavir	-0.051	-0.639	-0.207	0.106	0.524
Steroids	0.138	1.689	-0.023	0.299	0.093
Tracheostomy	0.323	4.012	0.164	0.483	<0.01
Surgeries	0.053	0.418	-0.195	0.301	0.676
Paralytics	0.299	2.800	0.088	0.510	0.006
Culture positive infection	0.373	6.910	0.266	0.480	<0.01

**Table 6:** Multiple linear regression for predictors of LOSH. LOSH, length of stay in hospital

Predictor variables	Unstandardized coefficients	Standardized coefficients	95% Confidence interval for odds ratio		p value
	B	Odds ratio	Lower bound	Upper bound	
Gastric complaints	-0.127	-1.638	-0.281	0.026	0.103
Fever on admission	0.121	1.290	-0.064	0.305	0.199
Hypotension	-0.022	-0.373	-0.139	0.095	0.709
Ventilation	-0.033	-0.349	-0.220	0.154	0.728
Pressers	0.027	0.306	-0.145	0.198	0.760
Dialysis	-0.049	-0.781	-0.174	0.075	0.436
Lopinavir/Ritonavir	0.075	0.944	-0.081	0.230	0.347
Steroids	-0.003	-0.040	-0.157	0.151	0.968
Tracheostomy	0.353	3.604	0.160	0.546	<0.01
Surgeries	0.107	0.715	-0.188	0.401	0.476
Paralytics	0.160	1.564	-0.042	0.362	0.119
Culture positive infection	0.333	5.567	0.215	0.451	<0.01

**Table 7:** Multiple linear regression for predictors of length of MV. MV, mechanical ventilation

Predictor variables	Unstandardized coefficients	Standardized coefficients	95% Confidence interval for odds ratio		p value
	B	Odds ratio	Lower limit	Upper limit	
Gastric complaints	-0.224	-2.933	-0.374	-0.073	0.004
Fever on admission	0.132	1.585	-0.032	0.297	0.115
Hypotension	0.018	0.318	-0.092	0.128	0.751
Ventilation	-0.067	-0.607	-0.283	0.150	0.545
Pressers	0.086	1.087	-0.070	0.242	0.278
Dialysis	0.061	1.092	-0.050	0.172	0.276
Lopinavir/Ritonavir	-0.001	-0.008	-0.154	0.152	0.993
Steroids	0.100	1.248	-0.058	0.258	0.214
Tracheostomy	0.321	3.997	0.163	0.480	<0.01
Surgeries	0.113	0.902	-0.134	0.360	0.368
Paralytics	0.201	1.688	-0.034	0.436	0.093
Culture positive infection	0.340	6.303	0.233	0.446	<0.01

## Discussion

In this study, we recorded the occurrence of culture-proven secondary infections and analyzed their impact on clinical outcomes. The incidence of secondary infections was high, more than half of the patients experienced at least one coinfection during the ICU stay. Existing literature reported incidence ranging from 10% to 45% [9-11]. Our sample includes 84 episodes of bacteremia, 72 positive respiratory cultures, and 36 urinary tract infections (UTIs) (Table 3).

A review of the literature reveals that the incidence of secondary bacterial pulmonary infections in hospitalized COVID-19 patients is about 16% (4.8%-42.8%), whereas the incidence of secondary fungal infections in hospitalized COVID-19 patients is 6.3% (0.9%-33.3%) in observational studies [6,12]. Our results are similar to published data.

The three most commonly cultured organisms in patients with VAP were Klebsiella, Pseudomonas, and Staphylococcus aureus (Table 3). Observational studies also reported similar pattern; *Pseudomonas aeruginosa* (21.1%), Klebsiella species (17.2%), and *Staphylococcus aureus* (13.5%) [13]. We have only one case of Aspergillus in contrast to other studies which reported a higher occurrence [14]. Most likely because we did not perform bronchoalveolar lavage (BAL) on a routine basis.

In our sample, the rate of infection with multidrug resistant (MDR) organisms was about 23% and the majority of them were extended spectrum beta-lactamases (ESBL) producing Gram-negative bacilli; the most prevalent was Klebsiella species.

Our results documented that although the patients with SNIs are associated with increased mortality, considering all other confounding factors, SNIs do not predict mortality. Vijay et al. reported 68% mortality in patients with SNIs among patients admitted to ICUs of 10 community hospitals [15]. This difference most likely results from an overestimation of mortality in their cohort by ignoring significant confounders' contribution to mortality. Another possibility is that patients who developed a rapid course of illness who died within a shorter time frame, not long enough to have objective evidence for SNI may have mitigated the impact of SNI on mortality. In our institution, the intensivists have received additional training in recognizing and treating infections in the critical care setting in a timely manner, unlike in other institutions where there is a delay in diagnosis and request for infectious disease (ID) consultation [16]. The difference in the contribution of SNI to mortality between our institution and others may have been due to differences in standards for infection control despite similar patient loads [16]. Another reason could be that the prevalent use of empiric antibiotics might have produced selective pressure on sensitive bacteria and favored MDR bacteria.

We analyze the factors associated with mortality and found SNI is associated with significantly increased days of MV and stay in ICU and hospital. However, we cannot establish a cause-and-effect relationship between the duration of intermittent mandatory ventilation (IMV) or intensive care unit length of stay (ICU LOS) and the risk of infection. Furthermore, in the logistic regression model, SNI does not predict mortality.

Patients with higher BMI had a worse outcome in our sample. Similar results were found by others [17]. We also found continuous renal replacement therapy (CRRT) and the use of vasopressors predict higher mortality. Gupta et al. also documented higher than 60% mortality in patients with COVID-19 who require CRRT [18]. Our sample also reveals lower mortality in patients treated with steroids and tracheostomy. The steroid effect has been documented by a metanalysis in >30 studies on the issue [19]. A meta-analysis of >35 studies did not show any significant mortality benefits [20]. We believe the difference in our results may be from the difference in methods and type of procedure as all our patients (small sample) underwent after three weeks of invasive MV after two negative polymerase chain reaction (PCR) tests and strictly in operation theater by experienced dedicated otolaryngologist; therefore, our results may not be applicable to the general population.

Our study has several limitations. Our study was retrospective with a small sample size involving predominantly young males that cannot compare with robust data from prospective studies with much larger sample sizes. Antibiotics prescription was the decision of the treating physician. There was no multidisciplinary team with a pharmacist or infectious disease consultation which can always be helpful. We included in the analysis exclusively microbiologically confirmed infections; we may have underestimated the incidence of infectious episodes, neglecting some difficult-to-diagnose infections (e.g., invasive aspergillosis). Most culture samples were blind respiratory tracheal aspirate as we did not perform bronchoalveolar lavage. Some studies have reported the incidence of secondary fungal pulmonary infections as about 20% when bronchoscopy with BAL was performed routinely post-intubation [21-22]. Finally, we studied only COVID-19 patients, therefore, we cannot draw any conclusion about a causal association between COVID-19 and an increased risk of SNI.

## Conclusions

Secondary non-viral infections are highly prevalent in patients admitted to ICU for COVID-19 ARDS. Although patients with SNIs have higher rates of mortality than patients without but they do not seem to significantly predict mortality in our sample. Secondary non-viral infections may prolong the days of MV, LOSICU, and LOSH. Prevalence of infection with MDR bacteria among patients with COVID-19 infections admitted to ICU is high and mostly exhibits extended-spectrum beta-lactamases. 
